# Iron Overload Favors the Elimination of *Leishmania infantum* from Mouse Tissues through Interaction with Reactive Oxygen and Nitrogen Species

**DOI:** 10.1371/journal.pntd.0002061

**Published:** 2013-02-14

**Authors:** Sílvia Vale-Costa, Sandra Gomes-Pereira, Carlos Miguel Teixeira, Gustavo Rosa, Pedro Nuno Rodrigues, Ana Tomás, Rui Appelberg, Maria Salomé Gomes

**Affiliations:** 1 IBMC - Instituto de Biologia Molecular e Celular, Universidade do Porto, Porto, Portugal; 2 ICBAS - Instituto de Ciências Biomédicas de Abel Salazar, Universidade do Porto, Porto, Portugal; 3 CISA-ESTSP - Núcleo de Investigação em Farmácia, Centro de Investigação em Saúde e Ambiente, Escola Superior de Tecnologia da Saúde do Porto, Instituto Politécnico do Porto, Porto, Portugal; Instituto Oswaldo Cruz, Brazil

## Abstract

Iron plays a central role in host-parasite interactions, since both intervenients need iron for survival and growth, but are sensitive to iron-mediated toxicity. The host's iron overload is often associated with susceptibility to infection. However, it has been previously reported that iron overload prevented the growth of *Leishmania major*, an agent of cutaneous leishmaniasis, in BALB/c mice. In order to further clarify the impact of iron modulation on the growth of *Leishmania in vivo*, we studied the effects of iron supplementation or deprivation on the growth of *L. infantum*, the causative agent of Mediterranean visceral leishmaniasis, in the mouse model. We found that dietary iron deficiency did not affect the protozoan growth, whereas iron overload decreased its replication in the liver and spleen of a susceptible mouse strain. The fact that the iron-induced inhibitory effect could not be seen in mice deficient in NADPH dependent oxidase or nitric oxide synthase 2 suggests that iron eliminates *L. infantum in vivo* through the interaction with reactive oxygen and nitrogen species. Iron overload did not significantly alter the mouse adaptive immune response against *L. infantum*. Furthermore, the inhibitory action of iron towards *L. infantum* was also observed, in a dose dependent manner, in axenic cultures of promastigotes and amastigotes. Importantly, high iron concentrations were needed to achieve such effects. In conclusion, externally added iron synergizes with the host's oxidative mechanisms of defense in eliminating *L. infantum* from mouse tissues. Additionally, the direct toxicity of iron against *Leishmania* suggests a potential use of this metal as a therapeutic tool or the further exploration of iron anti-parasitic mechanisms for the design of new drugs.

## Author Summary

*Leishmania* are important vector-borne protozoan pathogens that cause different forms of disease, ranging from cutaneous self-healing lesions to life-threatening visceral infection. *L. infantum* is the most common species causing visceral leishmaniasis in Europe and the Mediterranean basin. Iron plays a critical role in host-pathogen interactions. Both the microorganism and its host need iron for growth. However, iron may promote the formation of toxic reactive oxygen species, which contribute to pathogen elimination, but also to host tissue pathology. We investigated the effect of manipulating host iron status on the outcome of *L. infantum* infection, using the mouse as an experimental model. We found that dietary iron deprivation had no effect on *L. infantum* growth, and iron-dextran injection decreased the multiplication of *L. infantum* in mouse organs. The fact that this anti-parasitic effect of iron was not observed in mice genetically deficient in superoxide and nitric oxide synthesis pathways indicates that iron is likely to act in synergy with reactive oxygen and nitrogen species produced by the host's macrophages. This work clearly shows that iron supplementation improves the host's capacity to eliminate *L. infantum* parasites and suggests that iron may be further explored as a therapeutic tool to fight this type of infection.

## Introduction

*Leishmania* are trypanosomatid protozoans that alternate between two forms: the extracellular motile promastigote in the gut of phlebotomine insects and the intracellular non-motile amastigote inside the macrophages of mammalian hosts. These parasites cause leishmaniasis, a spectrum of human diseases that range from self-healing cutaneous ulcers to fatal visceralizing infection. Every year, approximately 2.0 million people develop symptomatic disease (0.5 million of them the visceral form) [1]. In Europe, visceral leishmaniasis is caused almost exclusively by *L. infantum*, which is transmitted as a zoonosis. The domestic dog is one of the main reservoirs of this parasite and canine leishmaniasis is an important veterinary problem in European Mediterranean countries [2].

There are currently no effective vaccines to prevent human leishmaniasis [3]. Therefore, management of the disease relies on chemotherapy. However, available drugs are highly toxic and the frequency of resistant parasite strains is increasing worldwide [4], [5]. The improvement of our knowledge on the mechanisms of host resistance to *Leishmania* is important to contribute to the development of new therapeutic strategies.

The important role played by iron metabolism in the interaction between host and pathogens is being increasingly highlighted by recent research [6], [7]. Both the host and the pathogens absolutely need iron for survival and must have efficient mechanisms for its acquisition together with adequate mechanisms of cell defense to avoid iron toxicity. Data obtained in human patients, as well as in different animal infection models indicate that in many cases iron availability favors the multiplication of pathogens, whereas iron deprivation impairs their growth [8]. Interestingly, the vertebrates innate immune response to infection includes several mechanisms of iron with-holding such as lactoferrin, hepcidin, Nramp1 or lipocalin2 [6]. Still, adequate concentrations of iron are required to support macrophagic killing mechanisms during infection [9], [10]. Contrary to what happens with other pathogens, the growth of *Brucella abortus* inside macrophages [11] and that of *L. major* in the mouse [12] are decreased by host's iron overload. In both cases, killing was correlated to oxidative burst [11], [13]. These examples highlight that iron can be exploited, in some cases, by the host to strengthen its antimicrobial defense mechanisms.

Our group has previously shown that the infection by *Mycobacterium avium*, an intra-macrophagic pathogen, is clearly exacerbated by host's iron overload, either genetically determined [14] or caused by iron-dextran injection [15], [16]. Furthermore, we have also demonstrated that iron chelation can be used to inhibit the growth of *M. avium* [17]. In the present work, we evaluated the effect of iron on the growth of *L. infantum*, the agent of European visceral leishmaniasis. We found that iron consistently inhibited the replication of *L. infantum* both by a direct effect and through the activity of the host's macrophage.

## Materials and Methods

### Animals and ethics statement

BALB/c and C57BL/6 mice were purchased from Charles River (Madrid, Spain). Mice deficient on the p47 subunit of the NADPH oxidase complex, on a C57BL/6 background (p47phox^−/−^), were bred at IBMC from a breeding pair purchased from Taconic (Lille Skensved, Denmark). The p47phox^−/−^ mice were administered trimethoprimsulfamethoxazole (Bactrim; 600 mgl^−1^) in the drinking water, as prophylactic treatment against bacterial infection. This treatment was ceased when infection experiments began. Mice deficient in the nitric oxide synthase 2, on a C57BL/6 background (NOS2^−/−^), were bred at IBMC from a breeding pair kindly provided by Drs. J. Mudgett, J. D. MacMicking and C. Nathan (Cornell University, New York, USA). Mice deficient in HFE, on a C57BL/6 background (Hfe^−/−^) were bred at IBMC from a breeding pair obtained from Centre Nationale de la Recherche Scientifique. All animals were housed at IBMC facilities under specific pathogen free conditions and fed *ad libitum*, except for the diet experiments indicated below. All animals were used at 8 to 16 weeks of age. Only female mice (average 20–25 g) were used for *in vivo* experiments. Mice were euthanized by isofluorane anesthesia followed by cervical dislocation and tissues were collected in aseptic conditions.

The experimental animal procedures were approved by the Local Animal Ethics Committee of IBMC and licensed by the Portuguese General Directory of Veterinary (DGV, Ministry of Agriculture, Rural Development and Fishing), in May 18, 2006 with reference 520/000/000/2006. All animals were handled in strict accordance with good animal practice as defined by national authorities (DGV, Law nu1005/92 from 23^rd^ October) and European legislation EEC/86/609.

### Parasites

All experiments were performed with *L. infantum* strain MHOM/MA/67/ITMAP-263 (zymodeme MON-1). For each experiment, parasites were obtained from the spleens of infected mice. Promastigotes were differentiated from spleen amastigotes by culturing at 25°C in complete Schneider's medium (Sigma-Aldrich Co., St Louis, MO, USA) supplemented with 20% heat-inactivated fetal bovine serum (FBS), 100 Uml^−1^ penicillin, 100 µgml^−1^ streptomycin (all from Gibco, Life Technologies, Carlsbad, CA, USA), 2% human urine, 5 µgml^−1^ phenol red (Sigma) and 5 mM HEPES sodium salt (Sigma) pH 7.4. Promastigote cultures were expanded at 25°C, for a maximum of 5 passages, in RPMI 1640 GlutaMAX™-I medium (Gibco, Life Technologies), containing 20% FBS, 50 Uml^−1^ penicillin, 50 µgml^−1^ streptomycin and 25 mM HEPES sodium salt pH 7.4. Promastigote differentiation from the exponential to the stationary phase was promoted by culture at 25°C, without medium renovation, for 4 to 5 days.

Axenic amastigotes were derived from the above-mentioned *L. infantum* strain, by the culture at 37°C with 7% CO_2_ atmosphere, in a medium for axenic amastigotes supplemented with 2 mM L-glutamine (GlutaMAX™-I, Gibco, Life Technologies) and 20% FBS (MAA20 medium, adapted from [18]).

### Direct effect of iron on *Leishmania* promastigote and amastigote cultures

Promastigotes (2×10^6^/well) and amastigotes (4×10^5^/well) of *L. infantum* were cultured in complete RPMI (25°C) or MAA20 (37°C) medium, respectively, supplemented with iron-dextran (Fe^3+^ hydroxide-dextran complex, Sigma), iron citrate (Fe^3+^, Sigma) and iron sulphate (Fe^2+^, Merck) in the concentrations of 0.018, 0.035, 0.070, 0.14, 0.28, 0.56, 1.1, 2.2, 4.5, 9 and 18 mM (96 well plates). Equivalent concentrations of dextran (Sigma), tri-sodium citrate (Merck) and magnesium sulphate (Merck) were used as controls. After 24 h of culture, 20 µl of a 2.5 mM resazurin solution (freshly prepared and filtered in phosphate buffered saline, pH 7.4, Sigma) was added to each well. The fluorescence intensity (excitation at 560 nm and emission at 590 nm) was determined 24 h (for amastigotes) or 48 h (for promastigotes) after resazurin addition to allow conversion to fluorescent resorufin, with a fluorometer SpectraMAX GeminiXS (Molecular Devices LLC, Sunnyvale, CA). In order to exclude a possible interference of iron with resazurin conversion to resorufin, appropriate controls were included without cells. No resazurin conversion was detected in the absence of parasites. Complete RPMI and MAA20 medium contains approximately 6 and 7.7 µM of iron, respectively, according to supplier's information.

### Animal experimental infection and parasite burden quantification

Mice were injected in the lateral vein of the tail with 2×10^7^ *L. infantum* stationary promastigotes in 200 µl of phosphate buffered saline pH 7.4 (PBS). At defined time points, the animals were euthanized and total livers and spleens were removed and homogenized, respectively, in 3.5 ml and 3 ml of complete Schneider's medium. These suspensions were further diluted 1∶100 (liver) or 1∶10 (spleen). Four-fold serial dilutions of the homogenized tissue suspensions were performed in quadruplicate (96 well plates). After 7 to 14 days at 25°C, the wells were examined for viable promastigotes. The reciprocal of the highest dilution that was positive for parasites was considered to be the number of parasites per ml of suspension and was used to calculate the number of parasites per organ (parasite burden).

### Animal experimental iron overload

In the kinetics experiments, 1 mg of iron, as iron-dextran (Sigma), was administered every other day i.p. to each mouse, from day −20 to day −2 of infection (a total of 10 mg of iron per mouse). Control mice received equivalent amounts of dextran (Sigma) by the same route. Parallel studies allowed us to verify that the administration of 10 mg of iron in a single injection produced the same effect in terms of outcome of *Leishmania* infection and also that dextran alone had no effect on the course of infection. Consequently, in subsequent experiments, iron overload was achieved by one single injection with 10 mg of iron per mouse. Control animals were injected with saline solution pH 6.0. None of the iron-dextran doses tested were toxic to the mice, as treated but uninfected mice remained healthy.

### Animal experimental iron deprivation

Mice were fed iron-free chow (Mucedola, Milan, Italy), from weaning until the end of the experiment (24 weeks). Control mice were fed a chow that differed only at its iron content (180 mgKg^−1^, Mucedola, Milan, Italy). Chow was administered in plastic recipients to avoid metal contamination. Animals were allowed to drink deionized water.

### Tissue iron determination

Non-heme iron was measured in tissues by the bathophenanthroline method [19]. Briefly, tissue samples (30–100 mg) were weighted, placed in iron-free Teflon vessels (ACV-Advanced Composite Vessel, CEM Corporation, Matthews NC, USA) and dried in a microwave oven (MDS 2000, CEM Corporation). Subsequently, dry tissue weights were determined and samples digested in an acid mixture (30% hydrochloric acid and 10% trichloroacetic acid) for 20 h at 65°C. After digestion, a chromogen reagent (5 volumes of deionized water, 5 volumes of saturated sodium acetate and 1 volume of 0.1% bathophenanthroline sulfonate/1% thioglycollic acid) was added to the samples in order to react with iron and obtain a colored product that was measured spectrophotometrically at 535 nm. The extinction coefficient for bathophenanthroline is 22.14 mM^−1^cm^−1^. Iron content in tissues was expressed as µg non-heme iron/organ.

### Cytokine quantification

Liver (100 mg) and spleen (50 mg) samples were lysed in Bio-plex cell lysis buffer containing 2 mM phenylmethanesulfonyl fluoride (PMSF, Bio-Rad Laboratories Inc., CA, USA), sonicated for 5 min in a refrigerated bath and centrifuged at 4500 g for 4 min at 4°C to remove debris. Total protein was quantified in supernatants with the MicroBCA kit (Pierce, Thermo Fisher Scientific). Clear homogenates were diluted to 1.5 mgml^−1^ in PBS pH 7.4 containing 0.5% bovine serum albumin and again centrifuged at 16000 g for 10 min at 4°C. Cytokine quantification in liver and spleen homogenates was performed following Bio-plex assay (Bio-Rad) instructions.

### Flow cytometry analysis

Spleen cells were obtained by teasing these organs gently with forceps and incubating them in NH_4_Cl haemolytic buffer to lyse any remaining erythrocytes. Cell suspensions were then washed with HBSS and resuspended in DMEM/10% FBS.

For immunofluorescence staining, 10^6^ splenic cells were incubated for 15 min at 4°C, in a 96 well plate, with fluorescein isothiocyanate (FITC)-conjugated anti-DX5 (1∶200), anti-Gr1 (1∶800) or anti-CD19 (1∶200) antibodies, phycoerythrin (PE)-conjugated anti-CD3 (1∶200), anti-CD11c (1∶200) or anti-CD11b (1∶400) antibodies and allophycocyanin-conjugated anti-mouse F4/80 (1∶200) antibodies (BD Pharmingen, San Diego, CA, USA), in PBS containing 1% FBS in order to analyze spleen cell populations during infection. The cells were washed twice with PBS/1% FBS. The analysis of the cell populations was based on the acquisition of 10 000 events in a Becton Dickinson (BD, Franklin Lakes, NJ, USA) FACSCalibur equipped with BD CELLQuest and FlowJo (Tree Star Inc., Ashland, OR, USA) software.

### Histological analysis

Liver samples (50 mg) were fixated in 4% buffered paraformaldehyde pH 7.4 and embedded in paraffin. Tissue sections (5 µm) were stained with Perls' blue stain for iron detection. Representative pictures were obtained with an Olympus CX31 light microscope equipped with a DP-25 camera (Imaging Software CellˆB, Olympus, Center Valley, PA, USA).

### Statistical analysis

Statistical analysis was carried out using GraphPad Prism 5.0 software (GraphPad Software Inc., La Jolla, CA, USA). Student's *t*-test was used to estimate the statistical significance of the differences between groups. Multiple comparisons were performed with One-way ANOVA followed by Dunnett or Student Newman-Keuls *post hoc* test. Differences between groups were considered statistically significant when p value was less than 0.05 (*p<0.05; **p<0.01; ***p<0.001).

## Results

### Iron deprivation does not affect *L. infantum* growth in mouse tissues

**Figure 1:**
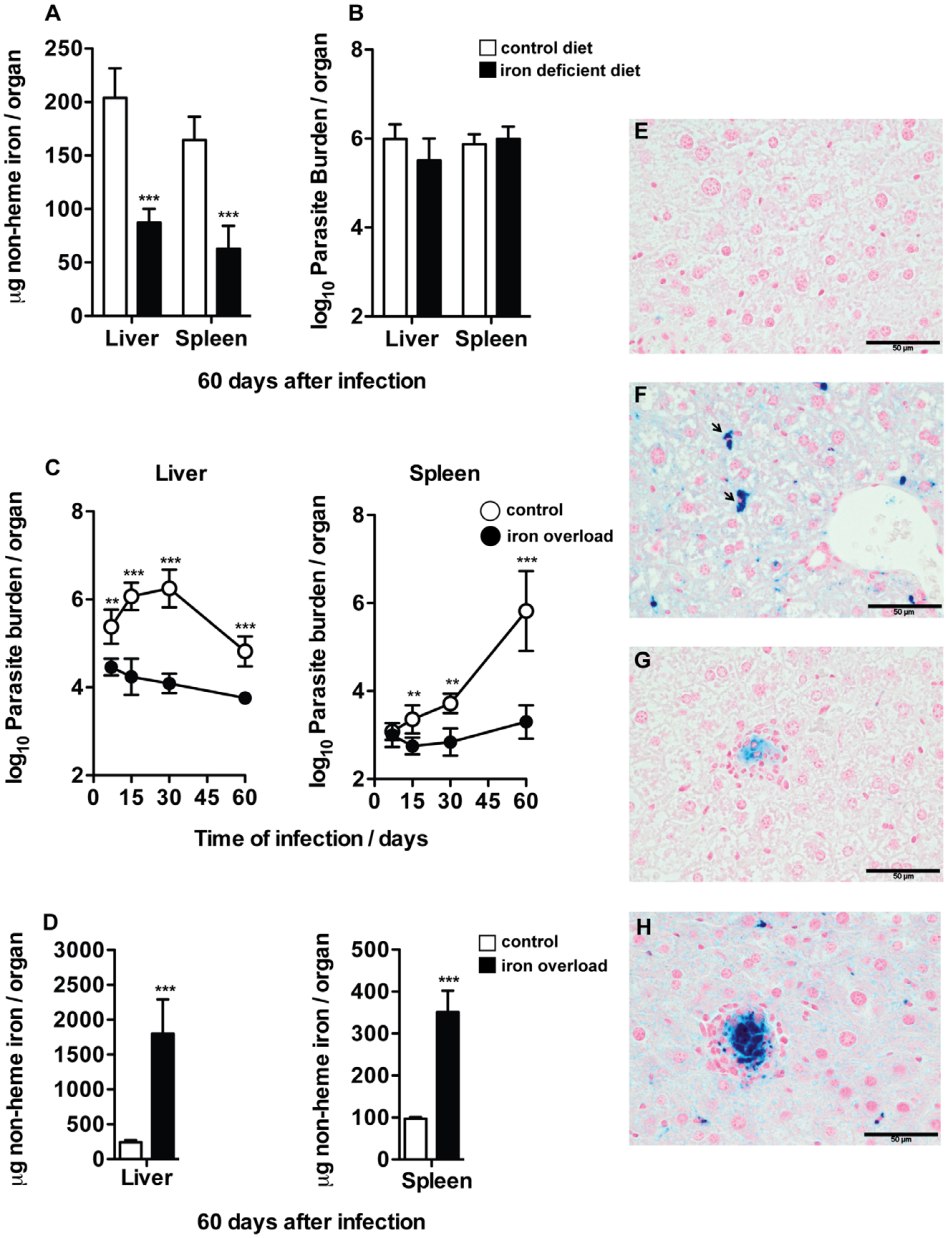
Effect of iron deprivation or overload on the growth of *L. infantum* in the mouse. A. B. BALB/c mice were kept on a control or iron deficient diet for 120 days prior to their i.v. infection with 2×10^7^ stationary promastigotes of *L. infantum*. Mice were sacrificed 60 days after infection. **A.** Non-heme iron content of liver and spleen was quantified in mice fed the control (white bars) or iron deficient diet (black bars). The graph shows the average+standard deviation of the non-heme iron content, expressed as µg/organ (*n* = 6). **B.** The parasite burden in the liver and spleen of groups fed the control (white bars) or iron deficient (black bars) diet was quantified by limiting dilution. The graph shows the average+standard deviation of the log_10_ number of parasites per organ (*n* = 6). **C.** BALB/c mice were i.p. injected with 1 mg of iron (given as dextran), in 10 alternate days, from day −20 to day −2 of infection (a total of 10 mg of iron per animal). Control mice received equivalent amounts of dextran by the same route. Mice were infected i.v. with 2×10^7^ stationary promastigotes of *L. infantum* and were sacrificed at 7, 15, 30 and 60 days of infection. The parasite burden in the liver and spleen of control (white circles) or iron overloaded (black circles) groups was quantified by limiting dilution (*n* = 5). **D.** Non-heme iron content in the liver and spleen was quantified in control (white bars) and iron overloaded (black bars) groups at 60 days after infection (*n* = 4–5). Student's *t*-test was performed to determine the statistical significance of the differences between groups (**p<0.01; ***p<0.001). **E. F. G. H.** Perl's blue staining was performed in liver sections of uninfected (**E**), uninfected iron overloaded (**F**), 60 days-infected (**G**) and 60 days-infected iron overloaded (**H**) mice. Black bar corresponds to 50 µm. The results of one representative experiment are shown. Two experiments were performed with similar results.

Iron withdrawal has been suggested as a means of controlling the growth of several unrelated pathogens [8], [20]. To evaluate the effect of iron deprivation on the growth of *L. infantum*, BALB/c mice were fed normal or iron-deficient chow for the first 120 days of life. They were subsequently infected and kept in the respective diets for the next 60 days, before being euthanized. Non-heme iron quantification in the liver and spleen confirmed that mice kept on an iron-deficient diet had less than half the amount of iron found in controls (Figure 1A). However, no differences in parasite load were observed between groups fed control or iron-deficient diets (Figure 1B), indicating that a mild iron deficiency has no impact on *L. infantum* replication in mouse tissues.

### Iron overload decreases the growth of *L. infantum* in the mouse tissues

Iron overload correlates with increased susceptibility to a great variety of pathogens [8], [20]. In order to determine the effect of host's iron overload on the infection by *L. infantum*, BALB/c mice were injected with 10 mg of iron-dextran or an equivalent amount of dextran before infection. Parasite burdens were determined in the livers and spleens at 7, 15, 30 and 60 days after infection. The growth of *L. infantum* in the liver and spleen of non-treated mice followed kinetics similar to that previously reported [21], [22]. During the first 30 days of infection, the parasite numbers increased significantly in both organs, returning to baseline levels in the liver thereafter, while continuing to grow in the spleen (Figure 1C). Conversely, parasite load was significantly lower in both organs of iron-overloaded mice throughout the experiment period (Figure 1C). Non-heme iron quantification confirmed that iron-overloaded animals had 8 and 3 times more iron, respectively, in the liver and spleen than control mice at 60 days after infection (Figure 1D). Iron distribution was analyzed in the liver, by Perl's staining. In those animals that were not injected with iron-dextran, iron deposition was very rarely seen (Figure 1E, G), the exception being the faint staining of some cell infiltrates (Figure 1G). The administration of iron to non-infected mice led to its accumulation predominantly in Kupffer cells (Figure 1F, black arrows), as expected from previous reports [23], [24]. Infection with *L. infantum*, led to the appearance of heavily iron-loaded macrophages inside cell infiltrates, the areas presumed to correspond to parasite containment (Figure 1H) [25], [26].

In order to investigate whether the *in vivo* anti-leishmanial effect of iron could be attributed to a generalized host tissue oxidative damage, we measured DNA cleavage and lipid peroxidation in the hepatic parenchyma through the immunofluorescence staining for TUNEL and 4-hydroxy-nonenal (4-HNE), respectively. Moreover, we assessed the formation of protein carbonyl groups in liver protein lysates by western blotting. However, we could not find any differences between control and iron-treated mice (Figures S1–S3 in Text S1), indicating that iron supplementation in our model did not cause a generalized oxidative damage to the tissue.

These experiments revealed that the accumulation of iron inside macrophages at *L. infantum* infection foci correlates with reduced parasite's multiplication, but not to generalized tissue damage.

### High concentrations of iron inhibit the axenic growth of *L. infantum*

In order to clarify the mechanisms by which iron exerts its anti-leishmanial effect in our model, we first asked whether iron could be exerting a toxic effect directly on the parasites.

**Figure 2:**
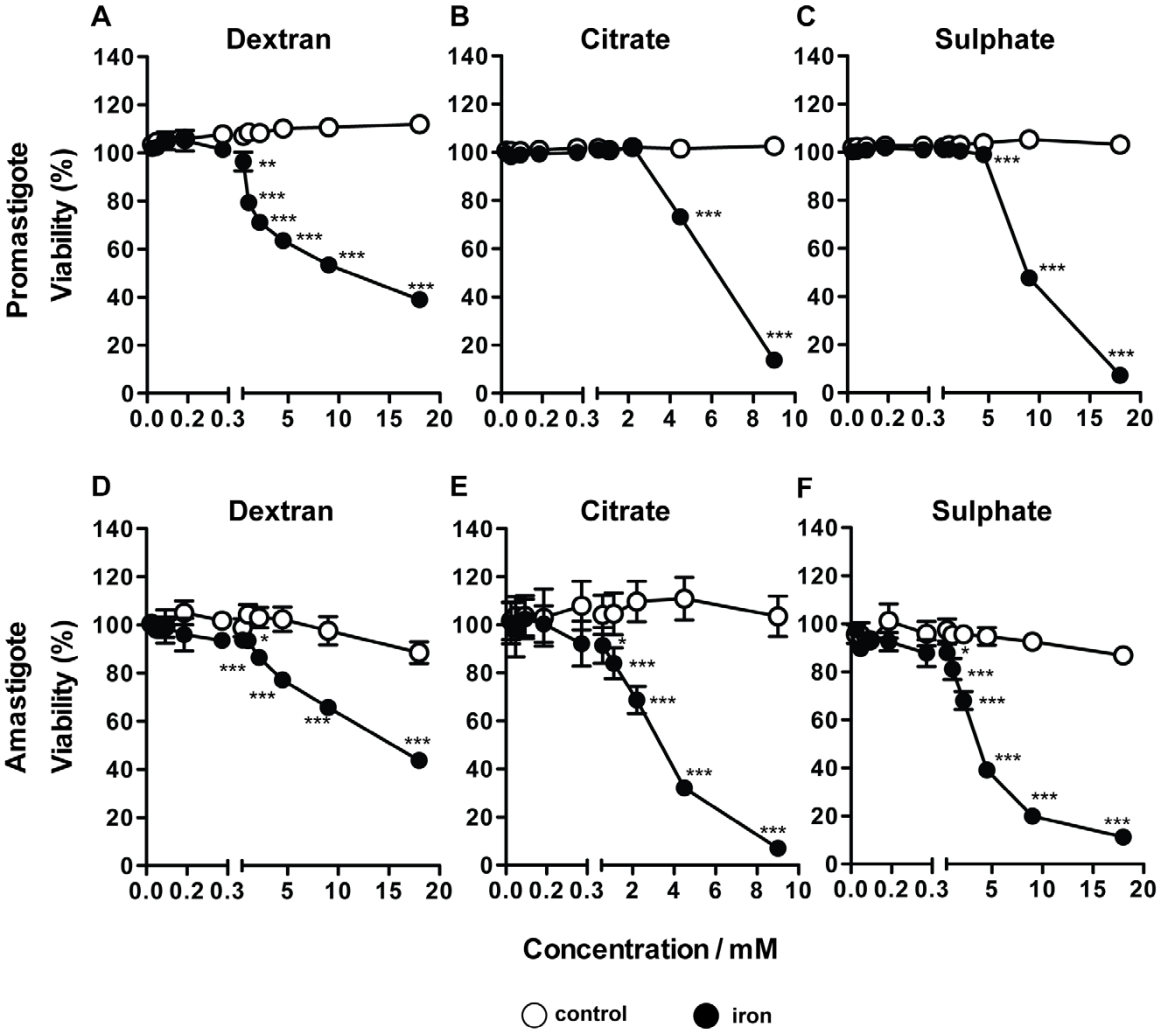
Effect of different forms of iron on the axenic growth of *L. infantum* pro- and amastigotes. Promastigotes in the exponential phase of growth (2×10^6^/well; **A, B, C**) or amastigotes (4×10^5^/well; **D, E, F**) were incubated in RPMI (25°C) or MAA20 (37°C) medium, respectively, with iron-dextran (**A**, **D**), iron citrate (**B**, **E**) and iron sulphate (**C**, **F**) (black circles) in the concentrations of 0.018, 0.035, 0.070, 0.14, 0.28, 0.56, 1.1, 2.2, 4.5, 9 and 18 mM. Equivalent concentrations of dextran (**A**, **D**), tri-sodium citrate (**B**, **E**) and magnesium sulphate (**C**, **F**) were used as controls (white circles). Resazurin dye was added after 24 h of culture. The fluorescence was measured 24 h (for amastigotes) or 48 h (for promastigotes) after resazurin addition. The results show the average ± standard deviation of the percentage viability in relation to the non-treated control (*n* = 3). One-way ANOVA, followed by a Newman-Keuls multiple comparison *post-hoc* test, was performed to determine the statistical significance of the differences between control (white circles) and iron treated (black circles) groups (*p<0.05, **p<0.01; ***p<0.001). The results of one representative experiment are shown. Three experiments were performed with similar results.

Axenic promastigotes (Figure 2A–C) or amastigotes (Figure 2D–F) of *L. infantum* were grown in the presence of increasing concentrations of iron (0.018–18 mM) in the form of either dextran (A,D) or citrate (B,E) complexes or sulphate (C,F) salt. *L. infantum* viability was measured, based on the parasite's capacity to metabolize the dye resazurin. Iron concentrations below 0.56 mM had no effect on the multiplication of the parasites (Figure 2). Amastigotes seemed to be more susceptible to iron toxicity, as iron decreased the viability of these parasites in a dose dependent manner from 0.56 to 18 mM irrespective of its molecular form (Figure 2D–F). Promastigotes were inhibited by iron-dextran (Fe^3+^) at 0.56 mM or above (Figure 2A), while iron citrate (Fe^3+^) and iron sulphate (Fe^2+^) were active against promastigotes only above 4.5 mM (Figure 2B, C). Promastigotes exposed to 9–18 mM of iron in any form displayed oval shape, atrophied cell body and reduced motility (not shown), changes which are characteristic of stress situations [27]. The results obtained by resazurin reduction were confirmed by the visual microscopic quantification of the parasites in a Neubauer chamber, on selected samples (not shown). Overall, these results indicate that iron can inhibit the growth or even kill *L. infantum* promastigotes and amastigotes, although the concentrations needed to achieve that effect are relatively high.

### Iron overload does not affect the capacity of the *L. infantum* infected mice to induce expansion of splenic cell populations or expression of key cytokines

Since previous studies had suggested that host's iron-overload interfered with the development of a protective immune response [12], we evaluated the impact of iron-overload on the induction of protective cytokines and specific splenic cell populations in our model of visceral leishmaniasis.

**Figure 3:**
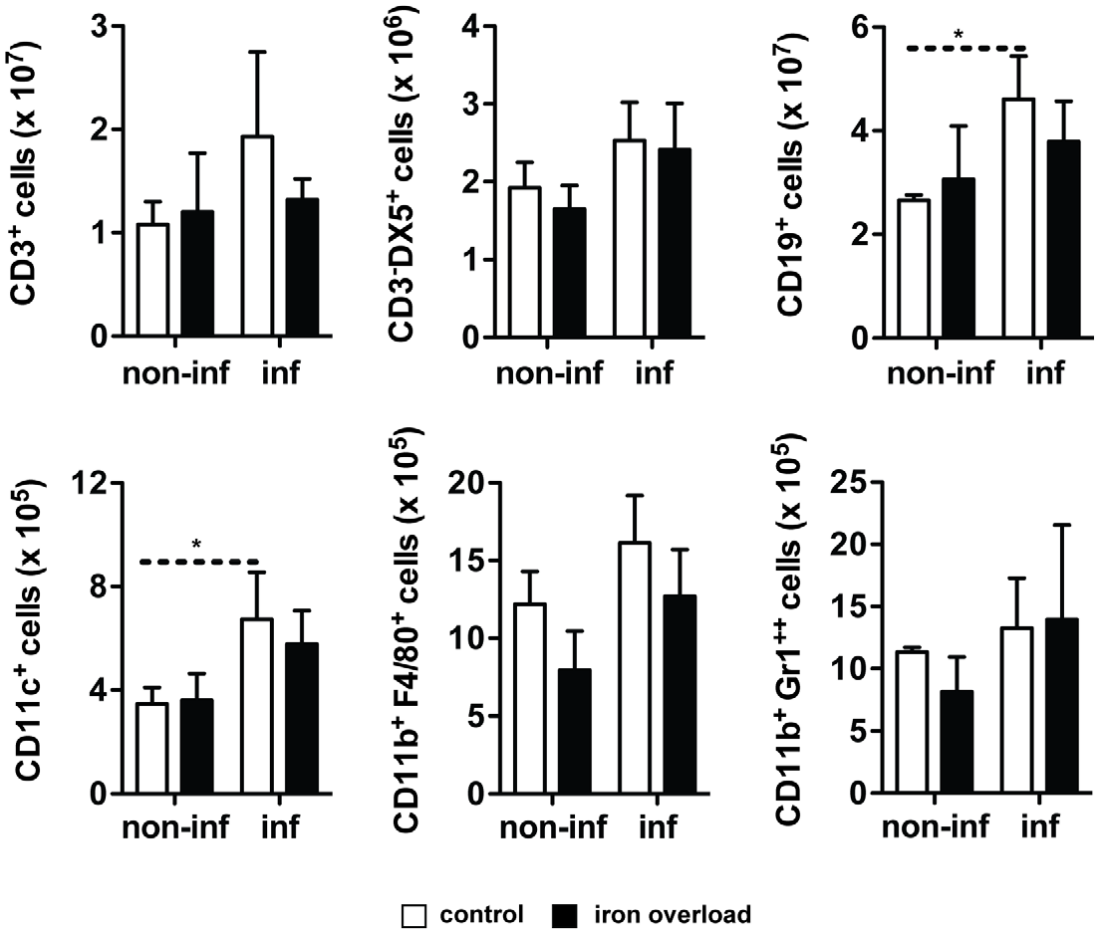
Effect of iron overload on the capacity of the mouse to induce the expansion of splenic cell populations. BALB/c mice were i.p. injected with saline solution or 10 mg of iron (-dextran, in a single dose) and were infected 15 days later by the i.v. route, with 2×10^7^ *L. infantum* stationary promastigotes (inf) or were left uninfected (non-inf). Mice were sacrificed 60 days later and the spleen was collected. The levels of CD3^+^, CD3^−^DX5^+^, CD19^+^, CD11c^+^, CD11b^+^F4/80^+^ and CD11b^+^GR1^++^ cells were quantified in the spleen of mice without (white bars) or with (black bars) iron overload. The results express the average+standard deviation of the total number of cells in the spleen (*n* = 3–5). One-way ANOVA, followed by a Newman-Keuls multiple comparison *post-hoc* test, was performed to determine the statistical significance of the differences between all groups (*p<0.05). The results of one representative experiment are shown. Two experiments were performed with similar results.

**Figure 4:**
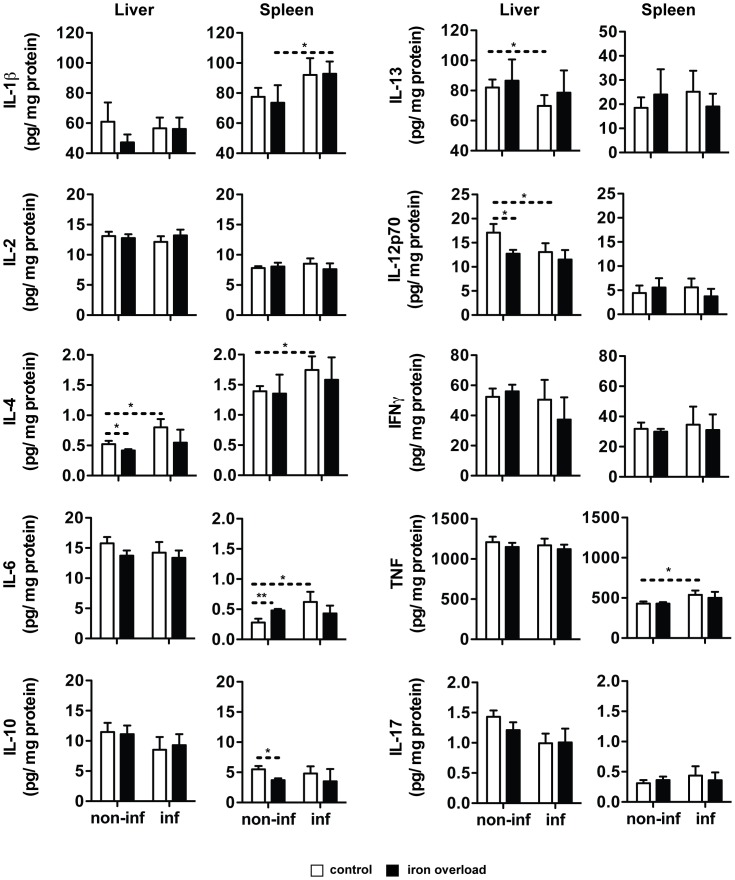
Effect of iron overload on the capacity of the mouse to induce the production of key cytokines. BALB/c mice were i.p. injected with saline solution or 10 mg of iron (-dextran, in a single dose) and were infected 15 days later by the i.v. route, with 2×10^7^ *L. infantum* stationary promastigotes (inf) or were left uninfected (non-inf). Mice were sacrificed 60 days later and liver (100 mg) and spleen (50 mg) samples were collected and homogenised in protein lysis buffer with 2 mM PMSF (Bio-plex Cell Lysis Kit, Biorad). The levels of IL (interleukin)-1β, IL-2, IL-4, IL-10, IL-13, IL-6, IL-12p70, IFNγ (interferon-gamma), TNF (tumour necrosis factor) and IL-17 were quantified in liver and spleen homogenates of mice without (white bars) or with (black bars) iron overload, accordingly to 10-Plex assay instructions (Biorad). The results express the average+standard deviation of the cytokine levels, expressed in pg/mg of protein (*n* = 3–5). Student's *t*-test was performed to determine the statistical significance of the differences between the different groups (*p<0.05; **p<0.01). The results of one representative experiment are shown.

BALB/c mice were treated with 10 mg of iron (given as iron-dextran) or saline solution 15 days prior to infection. They were infected with *L. infantum* and sacrificed 60 days later. Groups of non-infected mice were kept as controls. We performed a cytometric analysis of the number of splenic CD3^+^ (T cells), CD3^−^DX5^+^ (NK cells), CD19^+^ (B cells), CD11c^+^ (Dendritic cells), CD11b^+^F4/80^+^ (Macrophages) and CD11b^+^Gr1^++^ (Neutrophils) cells. The infection with *L. infantum* resulted in a significant increase in the numbers of CD19^+^ and CD11c^+^ cells, while other splenic cell sub-sets remained unaltered (Figure 3). More importantly, the number of cells belonging to each of the abovementioned populations was the same in control and iron overloaded groups (Figure 3). Additionally, the *in situ* production of a number of cytokines was measured, using a multiplex assay. The results of this screening revealed that infection with *L. infantum* did not have a dramatic impact on cytokine production. Only IL-1β, IL-6, TNF and IL-4 were significantly induced by infection in the spleen (the latter also in the liver, Figure 4), while the production of IL-12p70 and IL-13 decreased with infection, in the liver (Figure 4). No significant differences were found between iron-overloaded and control infected animals in any of the cytokines tested (Figure 4). The determination of cytokine mRNA expression in the tissues at earlier time-points did not reveal any differences between iron-overloaded and control infected mice (not shown).

Overall, these experiments indicate that the inhibitory effect of iron on the growth of *L. infantum* in the mouse does not result from an improvement of the activation of protective cells or increased production of protective cytokines.

### High iron doses and administration prior to infection are necessary for the inhibitory effect of iron

In order to further understand the mechanisms of the iron inhibitory effect on *L. infantum*, we tested the importance of different experimental parameters.

**Figure 5:**
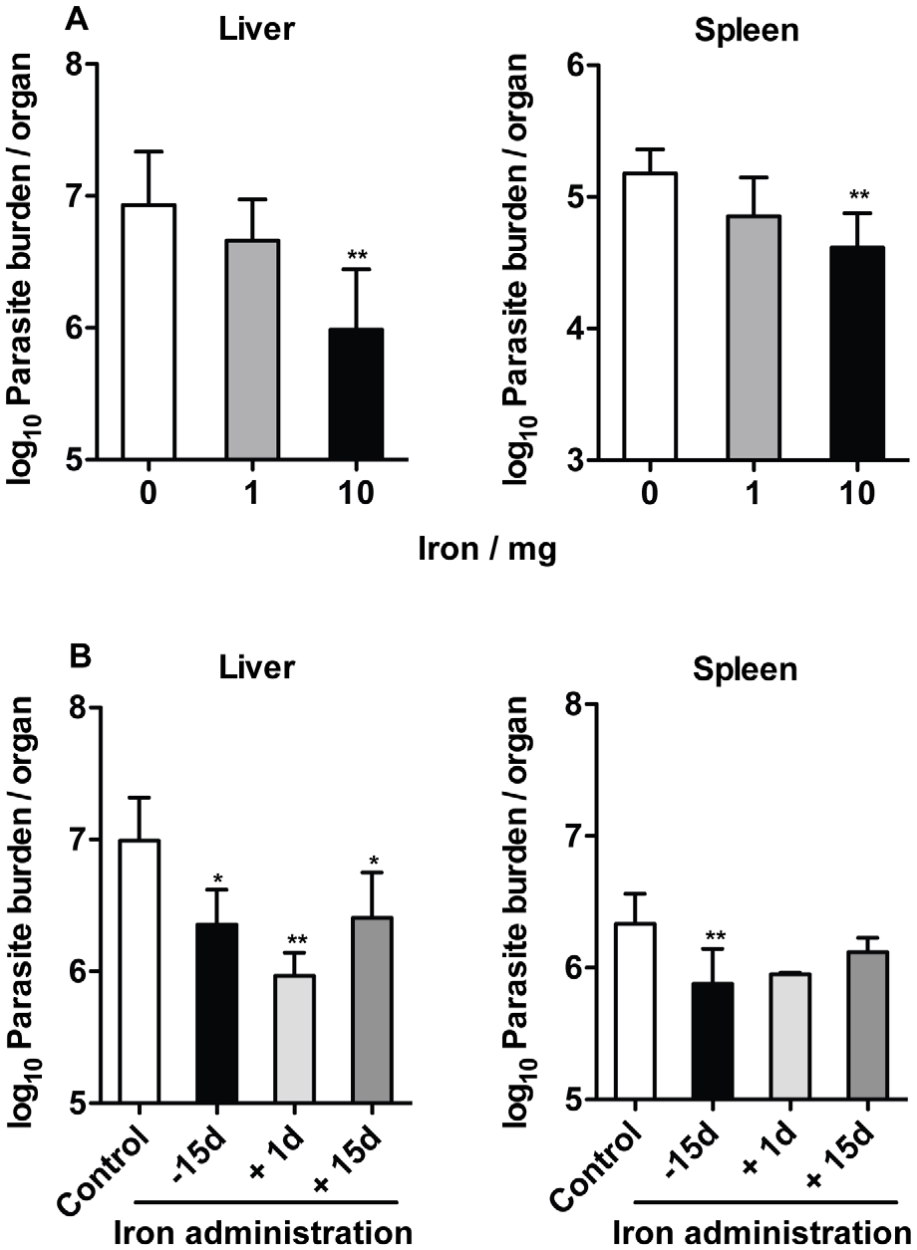
Effect of different iron doses and periods of administration on the growth of *L. infantum*. A. BALB/c mice were injected i.p. with 1 or 10 mg of iron (-dextran), in a single dose, 12 days before the infection. **B.** BALB/c mice were injected i.p. with 10 mg of iron (-dextran), in a single dose, 15 days before or 1 and 15 days after the infection (−15, +1 and +15 days, respectively). **A. B.** Control mice received saline solution by the same route. Mice were sacrificed 30 (**A**) or 60 (**B**) days after the i.v. infection with 2×10^7^ stationary promastigotes of *L. infantum*. The parasite burden in the liver and spleen of mice without (white bar) and with (gray - black bars) iron administration was determined by limiting dilution. The data shows the average+standard deviation of the log_10_ number of parasites per organ (*n* = 5). One-way ANOVA, followed by a Dunnett's multiple comparison *post-hoc* test, was performed to determine the statistical significance of the differences between each of the iron treated groups and control group (*p<0.05; **p<0.01). The results of one representative experiment are shown. Two experiments were performed with similar results.

First, we decided to assess if the same protective effect could be obtained with lower iron doses. BALB/c mice were injected with different amounts of iron (given as iron-dextran complex) prior to infection with *L. infantum*. Parasite loads were determined 30 days after infection. A significant inhibitory effect on the growth of *L. infantum* in the liver and spleen was seen only in mice that received 10 mg of iron (Figure 5A).

Next, we asked whether iron would still have an inhibitory effect on *L. infantum* growth when given after infection. We administered 10 mg of iron (-dextran) or saline solution to BALB/c mice, 1 or 15 days after infection. Parasite loads were determined 60 days post-infection. In the liver, similar levels of growth inhibition were seen when iron was given either before, 1 day after or 15 days after infection (Figure 5B). However, in the spleen and in contrast with iron pre-loading, no significant reduction on the growth of *L. infantum* was detected when the administration of iron was done after infection (Figure 5B).

**Figure 6:**
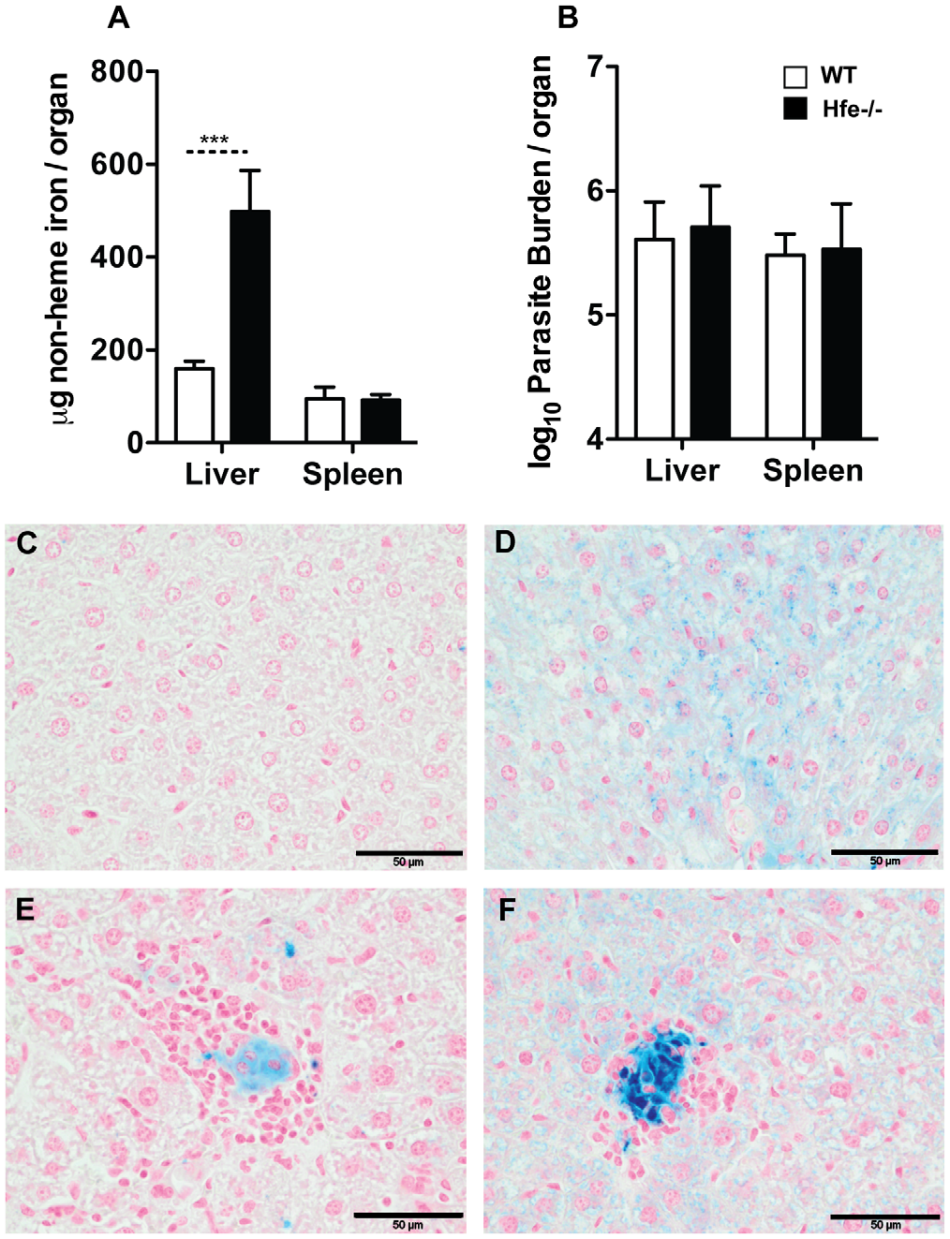
Effect of genetically determined iron overload on the growth of *L. infantum* in mice. **A. B.** Wild-type (WT) and Hfe^−/−^ mice were i.v. infected with 2×10^7^ stationary promastigotes of *L. infantum* and were sacrificed 60 days after infection. **A.** Non-heme iron content in both organs was quantified in WT (white bars) and Hfe^−/−^ (black bars) groups. The data shows the average+standard deviation of the non-heme iron content, expressed in µg/organ (*n* = 4–6). Student's *t*-test was performed to determine the statistical significance of the differences between WT and Hfe^−/−^ groups (***p<0.001). **B.** The parasite burden in the liver and spleen of wild-type (WT; white bars) and Hfe^−/−^ (black bars) mice was determined by limiting dilution (*n* = 5–6). **C–F:** Perl's blue staining was performed in liver sections of uninfected (**C**) and infected (**E**) WT and uninfected (**D**) and infected (**F**) Hfe^−/−^ mice. Black bar corresponds to 50 µm. The results of one representative experiment are shown. Two experiments were performed with similar results.

These results suggested that high amounts of iron present in the host prior to infection favor the decrease of the multiplication of *L. infantum*. In the model used, iron accumulation is observed in macrophages. To assess the relevance of the cellular location of iron deposition at the time of infection, we used HFE-deficient mice which are used as a model of human hemochromatosis and accumulate iron in parenchymal cells rather than inside macrophages [28], [29]. We infected Hfe^−/−^ mice with *L. infantum* and evaluated the parasite load and iron content in the livers and spleens at 60 days after infection. HFE-deficient mice had around 3 times more iron in the liver than controls and normal amounts of iron in the spleen (Figure 6A). As expected, in non-infected Hfe^−/−^ mice, iron was found predominantly in the hepatic parenchyma (Figure 6D). However, in *L. infantum*-infected mice, strong iron staining was found inside cell infiltrates both in wild-type mice and (more intensely) in Hfe^−/−^ mice (Figure 6E,F) showing that infection re-routed iron between the two cell types. Interestingly, the parasite loads in wild-type and Hfe^−/−^ mice were the same (Figure 6B).

Overall, these results indicate that a decrease in the growth of *L. infantum* is observed when high amounts of iron are found inside the host's macrophages, with a stronger effect when this occurs before the infection.

### Host production of reactive oxygen and nitrogen species is necessary for the inhibitory effect of iron overload on *L. infantum* growth

Iron concentrations necessary to inhibit *L. infantum* growth in axenic conditions are relatively high. So, we hypothesised that iron synergizes with antimicrobial mechanisms of macrophages, such as the production of reactive oxygen species (ROS) by the NADPH oxidase (respiratory burst) and reactive nitrogen species (RNS) by the nitric oxide synthase 2 (NOS2) to decrease *Leishmania* viability.

**Figure 7:**
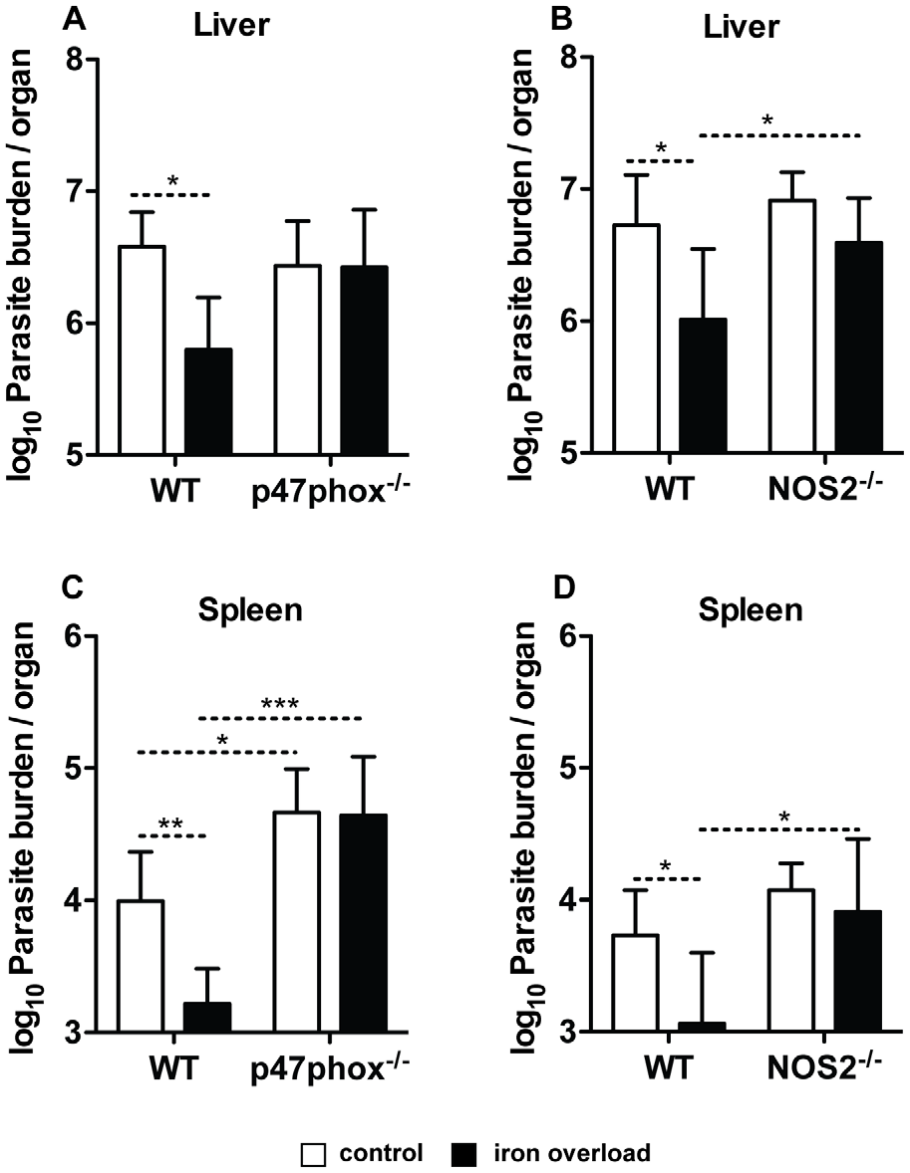
Effect of iron overload on the growth of *L. infantum* in mice: role of NADPH oxidase and NOS2. Wild-type (WT) and p47phox^−/−^ (**A**, **C**) or NOS2^−/−^ (**B**, **D**) mice were i.p. injected with saline solution or 10 mg of iron (-dextran, in a single dose) 15 days before i.v. infection with 2×10^7^ stationary promastigotes of *L. infantum* and were sacrificed 15 days later. The liver (**A**, **B**) and spleen (**C**, **D**) were removed and the parasite burden in mice without (white bars) or with (black bars) iron overload was quantified by limiting dilution. The results express the average+standard deviation of the log_10_ number of parasites per organ (*n* = 3–8). One-way ANOVA, followed by a Newman-Keuls multiple comparison *post-hoc* test, was performed to determine the statistical significance of the differences between all groups (*p<0.05; **p<0.01; ***p<0.001). The results of one representative experiment are shown. At least two experiments were performed with similar results.

Mice genetically deficient in the p47phox subunit of NADPH oxidase (p47phox^−/−^) and in the NOS2 enzyme (NOS2^−/−^) were used to test this hypothesis. Animals were treated with 10 mg of iron (-dextran) or saline solution and 15 days later were infected with *L. infantum*. Mice were sacrificed 15 days after infection and the parasite load was determined in the liver and spleen. Iron overload decreased *L. infantum* growth in wild-type but not in knock-out mice in the liver (Figure 7A, B) and spleen (Figure 7C, D), indicating that the mechanism through which iron exerts its inhibitory effect is dependent on the production of ROS and RNS by the host. A similar experiment in which animals were sacrificed 30 days after infection gave identical results (data not shown).

## Discussion

Iron is a central element in host-parasite interaction and several iron-depriving mechanisms are used by the host to inhibit pathogen proliferation [6], [7]. In contrast, previous work has shown that host's iron overload prevented the growth of *L. major* in BALB/c mice [12], [13]. In those studies, it is shown that iron overload correlates with increased production of ROS upon *L. major* infection [13], [30]. In the present work, we treated mice with iron-dextran and infected them with *L. infantum*, an agent of visceral leishmaniasis. We observed iron-loaded macrophages inside hepatic infiltrates, the areas of parasite containment, concomitantly with the decrease of tissue parasite loads. Such iron-associated decrease did not occur in p47phox- or NOS2-deficient mice, suggesting that iron exerts its effects through the combination with ROS and/or RNS produced by the macrophage. In fact, superoxide (O_2_•**^−^**) and nitric oxide (NO•), synthesized by the phagocytic NADPH oxidase and the nitric oxide synthase 2 (NOS2), respectively, have been implicated in the elimination of *Leishmania* by the host's macrophages [31], [32], [33], [34], [35], [36]. Moreover, both macrophagic NADPH oxidase and NOS2 require iron for proper function [37]. In a few models of macrophage infections with different bacteria, macrophages were shown to need iron to exert their antimicrobial activity. Iron increases the capacity of macrophages to eliminate or prevent the multiplication of *B. abortus*, by catalyzing the production of hydroxyl radical [11] and iron loading of *Staphylococcus aureus* prior to infection enhances bacterial killing by monocytes, most likely by promoting oxidative damage [38]. Also in the case of *Salmonella*, iron seems to be needed for intramacrophagic killing of these bacteria [10]. This suggests that iron, a well known pro-oxidant, promotes oxidative microbicidal mechanisms inside macrophages, to which *Leishmania* is sensitive [31], [33], [34], [35], [36], [39]. The fact that the anti-parasitic effect of iron is lost in mice deficient in only one of the two enzymes, either NOS2 or NADPH oxidase, suggests that both ROS and RNS are simultaneously required for iron to exert its anti-leishmanial effect. Iron can possibly favour the formation of peroxynitrite (ONOO^−^), a strong oxidizing species formed by the reaction of NO• with O_2_•**^−^** [40]. Since we could not find evidences of tissue oxidative damage (DNA damage, lipid peroxidation and protein oxidation) in iron-treated mice, we suggest that this formation of highly reactive ROS and RNS in combination with iron has a highly localized activity, inside the macrophage.

It was somewhat surprising that Hfe^−/−^ mice, which have spontaneous iron overload, predominantly in the liver, had tissue parasite loads similar to those of wild-type mice, when infected with *L. infantum*. This could be justified by the fact that Hfe^−/−^ mice develop spontaneous iron overload predominantly in hepatocytes, keeping macrophages relatively iron depleted [28], [29], [41]. When Hfe^−/−^ mice were infected with *L. infantum*, we could see iron accumulation inside macrophages at the infection foci, similarly to what we had previously reported in *M. avium* infection [14]. However, as suggested by the experiments in which we treated mice with iron-dextran after infection, the inhibitory effect of iron is best accomplished when the macrophages are iron-loaded prior to infection. Another hypothesis to explain the lack of an impact of Hfe^−/−^ iron overload on the growth of *L. infantum* is the level of iron overload in the tissues. Indeed, Hfe^−/−^ mice had tissue iron levels that were significantly lower than those found in iron-dextran-injected mice. The fact that even with iron-dextran injection, we needed high iron doses to decrease the parasite burden in tissues, argues for this hypothesis.

In the mouse model of cutaneous leishmaniasis, iron-induced respiratory burst at the onset of *L. major* infection is coupled to later activation of the nuclear transcription factor NF- κB [30] and to the display of a protective immune response [12]. Iron and ROS can modulate the activation of NF-κB signaling pathways [42], known to regulate several genes involved in immune and inflammatory responses [43]. In the case of *L. major* infection, mouse resistance is clearly related to an IL-12-driven, IFN-γ-dominated Th1 immune response, whereas susceptibility correlates with an IL-4-driven Th2 response [44]. Experimentally iron overloaded BALB/c mice, infected with *L. major*, exhibited a Th1-type immune response, with increased levels of IFNγ and NOS2 and decreased levels of IL-4 and IL-10 transcripts compared to untreated mice [12]. In accordance, supplementation of rats with iron-dextran [45] or saccharated colloidal iron [46] potentiated the induction of hepatic NOS2 and the production of NO• by LPS. However, the decrease of NO• production has also been observed in mice [47] and macrophages [48] treated with different iron sources. Additionally, delayed Th1 immune responses and Th2 phenotypes have been observed in response to iron supplementation in mice infected with *Cryptoccocus neoformans* [49] and *Candida albicans* [50], indicating that each particular host-pathogen interaction responds differently to iron overload.

In the case of visceral leishmaniasis, an efficient control of infection is also dependent on Th1 responses, although a mixed Th1/Th2 cytokine profile is detected during the course of infection [51], [52]. So, iron supplementation in our model, besides exerting a direct toxic effect on parasites in conjunction with ROS and RNS, could be improving the host's capacity to control the infection, by modulating the adaptive immune response. When we evaluated the cytokine response to *Leishmania* infection, we saw a discrete induction of IL-4 both in the liver and the spleen of infected mice, together with increases in the expression of the pro-inflammatory cytokines, IL-1β, IL-6 and TNF. However, iron overload did not significantly alter the immune response profile induced by infection, leading us to conclude that the modulation of the adaptive immune response does not contribute significantly to the protective effect of iron.

Malnutrition is associated with susceptibility to visceral leishmaniasis in humans [53], [54] and mice [55]. Although iron deficiency is the most common micronutrient deficiency in the human population [56], the relationship between human iron deficiency alone and increased risk of acquiring visceral leishmaniasis has never been investigated. In our model, feeding mice with an iron deficient diet did not affect *L. infantum* growth. These nutritionally iron-deprived animals had half the normal iron stores in the liver and the spleen, but presented normal haematocrit and body weight (not shown). We hypothesize that the low levels of iron in tissue stores were probably sufficient to maintain the growth of *Leishmania* and not low enough to impact on the host's capacity to control the infection. Observations regarding the effects of iron chelators on *Leishmania* growth are conflicting. Treatment of mice with desferrioxamine (DFO) led to the decrease of *L. infantum* proliferation [57] but not that of *L. major* [12]. Also, in *in vitro* models of macrophage infection, DFO has shown either no effect [58] or an inhibitory effect [59], [60], [61] on *Leishmania* growth. Finally, when tested on *Leishmania* promastigotes growing in culture medium, hydroxypiridinone-derived chelators showed an inhibitory effect, which was higher than that of DFO [62]. Thus, the available data do not allow inferring that *Leishmania* infections are amenable to treatment by iron depletion. It may be valuable to further explore the effects of different iron chelating ligands or of more drastic iron depletion protocols.

In addition to the results obtained *in vivo* and discussed above, we found axenic cultures of *L. infantum* to be sensitive to the direct toxicity of iron. It is plausible that high concentrations of iron (>0.56 mM) may have promoted the endogenous generation or propagation of reactive species in both parasite stages. On the other hand, lower iron doses (<0.56 mM) may not have been sufficient to overcome the antioxidant capacity of axenic *L. infantum*. In this regard, *L. infantum* promastigotes have been shown to accumulate iron in catalytically active forms, which contribute to their sensitivity to killing by hydrogen peroxide [63], [64], possibly through the Fenton reaction. Besides, upon exposure to high doses of this metal, *L. infantum* promastigotes exhibited impaired motility and morphological changes identical to those reported to occur after exposure to antimony (III) and arsenic (III) [27] (not shown). These metalloids, used for a long time as first line treatments against trypanosomatid infections, were recently found to act through the induction of oxidative damage in *L. donovani* [27], [65]. Interestingly, increased intracellular iron levels directly correlate to the parasite sensitivity to these drugs [27]. The influence of iron on anti-leishmanial drug activity also includes non-metalloid drugs. Iron potentiates the leishmanicidal activity of artemisinin [66], by inducing oxidative injury that culminates in cell death of *L. donovani* promastigotes. Furthermore, iron treatment can induce accumulation of pentamidine in the mitochondria of *L. enriettii* promastigotes, hence increasing their sensitivity to the drug. This effect is probably due to the action of the multidrug resistance protein 1 (LeMDR1), a putative mitochondrial iron importer [67]. Hence, the increase of intracellular iron levels in *Leishmania* overall increases its vulnerability to chemotherapy.

The interaction between pathogens and their hosts are complex processes dependent not only on the genome of both, but also on nutritional factors. In most of the reported cases, iron excess increases and iron chelation decreases susceptibility to infection [8], [20]. However, this is not observed in murine models of infection by *Leishmania*. Moreover, despite several reports that iron supplementation (to correct nutritional iron deficiency) can significantly increase the risk of several infections [8], [56], no correlation between iron administration and susceptibility to human leishmaniasis has, to our knowledge, ever been described. Although iron chelation has been suggested as an effective therapeutic strategy against several infections, in the case of leishmaniasis and especially in areas where this disease and malnutrition coexist, iron chelation may be inappropriate. Iron overload decreases *Leishmania* proliferation and induces parasite death, probably by promoting oxidative reactions pernicious to the parasite. The further investigation of the molecular mechanisms of these effects will be fundamental to explore a potential utilization of iron itself as a therapeutic tool and also to understand and improve the mechanisms of action of other anti-leishmanial drugs.

## Supporting Information

Text S1 **Effect of iron overload on the carbonylation of proteins, peroxidation of lipids and integrity of DNA in the mouse liver. Figure S1.** BALB/c mice were i.p. injected with saline solution or 10 mg of iron (-dextran, in a single dose) and were infected 15 days later by the i.v. route, with 2×10^7^ *L.infantum* stationary promastigotes. Mice were sacrificed 60 days later and liver (100 mg) samples were collected and homogenised in protein lysis buffer. Liver protein lysates were reacted with DNP-hydrazone and then separated by SDS-PAGE followed by Western blotting (*n* = 4–5). No differences in the protein carbonyl levels were observed between control and iron-treated mice. **Figure S2.** C57BL/6 mice were fed a control (**A**) or a 2.5% iron-carbonyl diet (**B**) for 15 days. Liver samples were assayed by immunofluorescence to detect 4-hydroxynonenal (4-HNE) staining (green). Nuclei were counterstained with DAPI (blue). No 4-HNE staining was detected in the liver tissue of BALB/c mice treated for 15 days with saline solution or 10 mg of iron-dextran prior to a 30- and 60-day infection with *L.infantum* (staining was identical to **A**). **Figure S3.** BALB/c mice were i.p. injected with saline solution (**A**) or 10 mg of iron (**B**) (-dextran, in a single dose) and were infected 15 days later by the i.v. route, with 2×10^7^ *L.infantum* stationary promastigotes. Mice were sacrificed 60 days later and liver samples were assayed by immunofluorescence to detect TUNEL staining (green). Nuclei were counterstained with DAPI (blue). No TUNEL staining was observed in animals receiving saline solution or iron treatment, except in the positive control (**C**, sample treated with DNase I).
(DOCX)
