# Supplementary material for: Iron Overload Favors the Elimination of Leishmania infantum from Mouse Tissues through Interaction with Reactive Oxygen and Nitrogen Species
Source: PLoS Negl Trop Dis. 2013 Feb 14;7(2):e2061. doi: 10.1371/journal.pntd.0002061 (PMC3573095; doi:10.1371/journal.pntd.0002061)
Supplement: Text S1 — Effect of iron overload on the carbonylation of proteins, peroxidation of lipids and integrity of DNA in the mouse liver. Figure S1. BALB/c mice were i.p. injected with saline solution or 10 mg of iron (-dextran, in a single dose) and were infected 15 days later by the i.v. route, with 2×10^7^ L.infantum stationary promastigotes. Mice were sacrificed 60 days later and liver (100 mg) samples were collected and homogenised in protein lysis buffer. Liver protein lysates were reacted with DNP-hydrazone and then separated by SDS-PAGE followed by Western blotting (n = 4–5). No differences in the protein carbonyl levels were observed between control and iron-treated mice. Figure S2. C57BL/6 mice were fed a control (A) or a 2.5% iron-carbonyl diet (B) for 15 days. Liver samples were assayed by immunofluorescence to detect 4-hydroxynonenal (4-HNE) staining (green). Nuclei were counterstained with DAPI (blue). No 4-HNE staining was detected in the liver tissue of BALB/c mice treated for 15 days with saline solution or 10 mg of iron-dextran prior to a 30- and 60-day infection with L.infantum (staining was identical to A). Figure S3. BALB/c mice were i.p. injected with saline solution (A) or 10 mg of iron (B) (-dextran, in a single dose) and were infected 15 days later by the i.v. route, with 2×10^7^ L.infantum stationary promastigotes. Mice were sacrificed 60 days later and liver samples were assayed by immunofluorescence to detect TUNEL staining (green). Nuclei were counterstained with DAPI (blue). No TUNEL staining was observed in animals receiving saline solution or iron treatment, except in the positive control (C, sample treated with DNase I). (DOCX) [file pntd.0002061.s001.docx]

##### **Text S1**


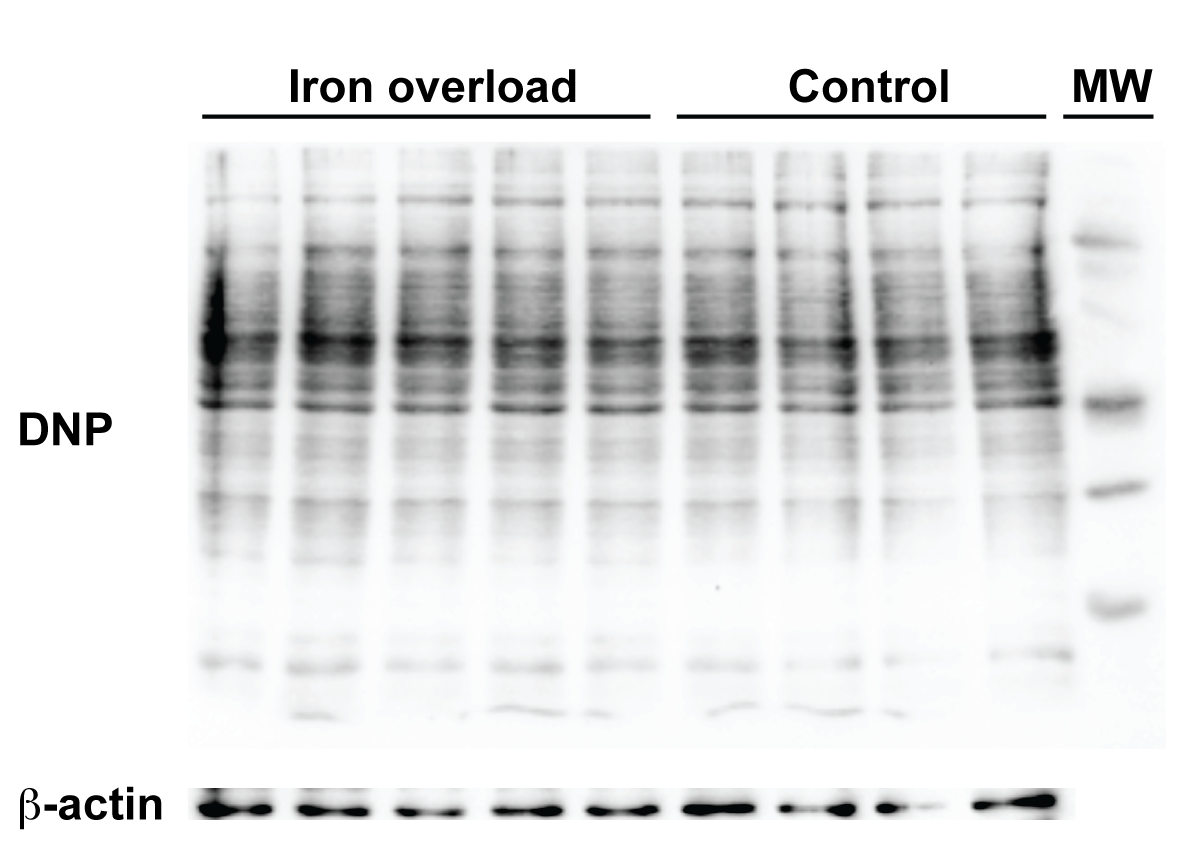


**Figure S1. Effect of iron overload on the carbonylation of proteins present in the liver homogenates of *L.infantum* infected mice.** BALB/c mice were i.p. injected with saline solution or 10 mg of iron (-dextran, in a single dose) and were infected 15 days later by the i.v. route, with 2 × 10^7^ *L.infantum* stationary promastigotes. Mice were sacrificed 60 days later and liver (100 mg) samples were collected and homogenised in protein lysis buffer. Liver protein lysates were reacted with DNP-hydrazone and then separated by SDS-PAGE followed by Western blotting (*n*=4-5). No differences in the protein carbonyl levels were observed between control and iron-treated mice.

#####
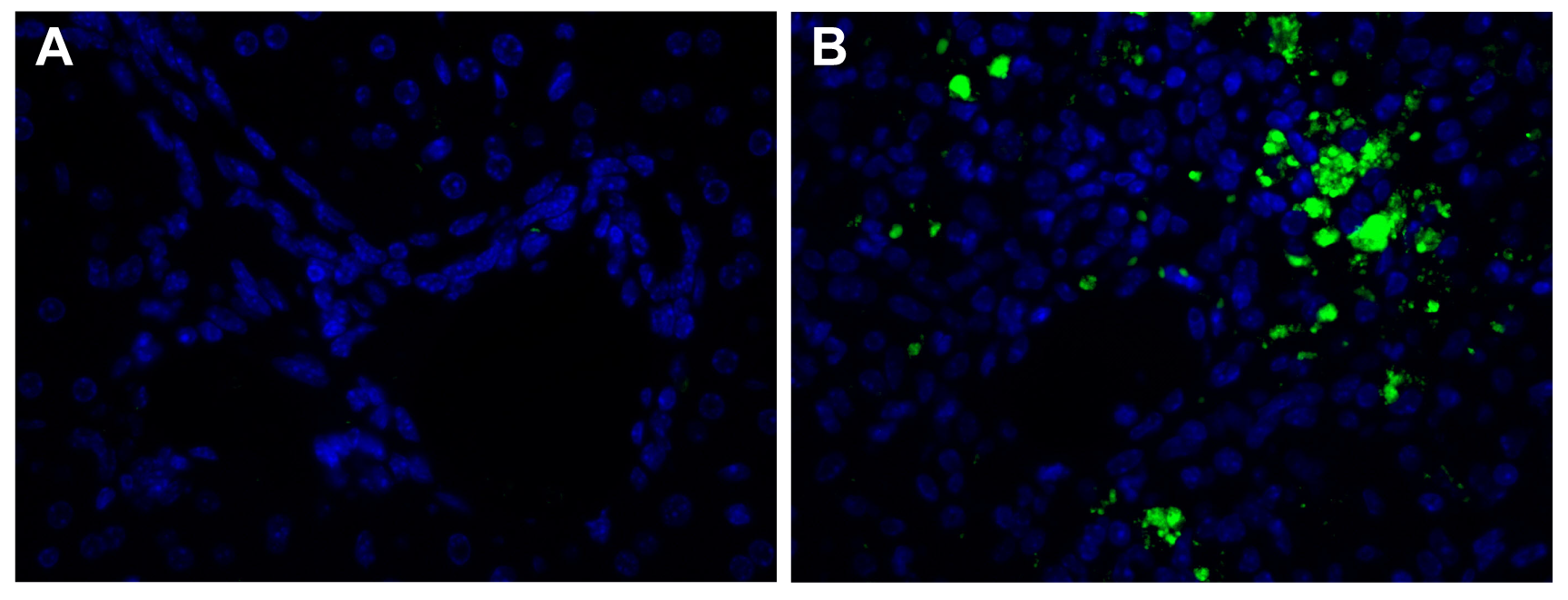


**Figure S2. Effect of iron overload on the lipid peroxidation of the mouse liver.** C57BL/6 mice were fed a control (**A**) or a 2.5% iron-carbonyl diet (**B**) for 15 days. Liver samples were assayed by immunofluorescence to detect 4-hydroxynonenal (4-HNE) staining (green). Nuclei were counterstained with DAPI (blue). No 4-HNE staining was detected in the liver tissue BALB/c mice treated for 15 days with saline solution or 10 mg of iron-dextran prior to a 30- and 60-day infection with *L.infantum* (staining was identical to **A**).

**
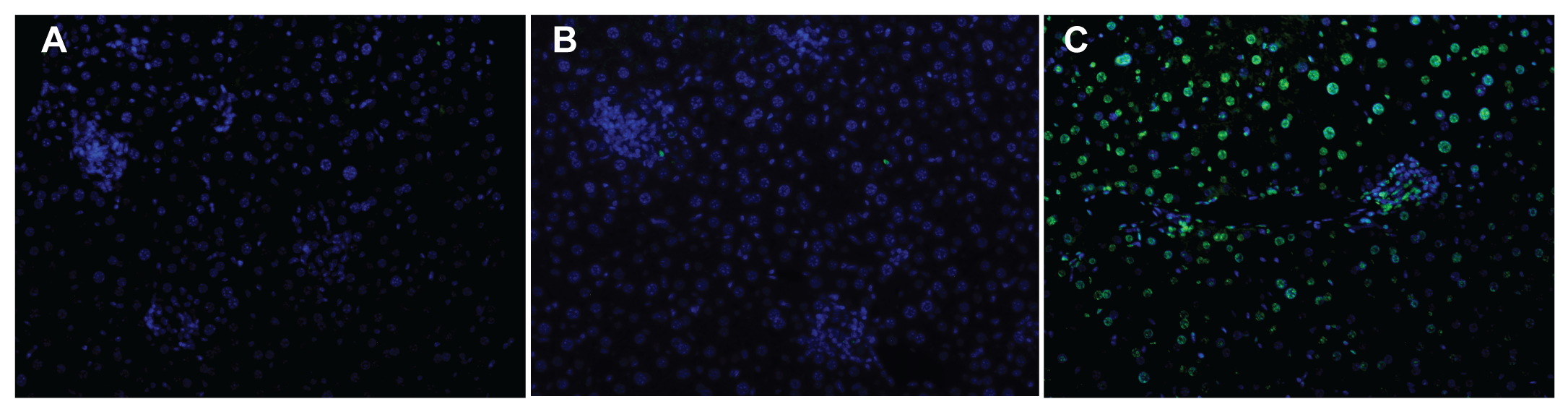
**

**Figure S3. Effect of iron overload on the integrity of DNA from the mouse liver.** BALB/c mice were i.p. injected with saline solution (**A**) or 10 mg of iron (**B**) (-dextran, in a single dose) and were infected 15 days later by the i.v. route, with 2 × 10^7^ *L.infantum* stationary promastigotes. Mice were sacrificed 60 days later and liver samples were assayed by immunofluorescence to detect TUNEL staining (green). Nuclei were counterstained with DAPI (blue). No TUNEL staining was observed in animals receiving saline solution or iron treatment, except in the positive control (**C,** sample treated with DNase I).

**Materials and methods**

**Protein carbonyl determination**

##### Liver samples (100 mg) were lysed in RIPA buffer containing 1% protease inhibitors (Sigma). Homogenates were centrifuged at 16000g for 10 min at 4ºC to remove debris. Total protein was quantified in supernatants with the Biorad DC Protein Assay kit (Biorad). The detection of protein oxidation was performed accordingly to the instructions of the OxyBlot Protein Oxidation Detection kit (Millipore, Billerica, MA, USA). Briefly, the carbonyl groups in liver protein samples (15 μg) were first derivatized to 2,4-dinitrophenylhydrazone (DNP) by reaction with 2,4-dinitrophenylhydrazine (DNPH), in the presence of 6% SDS. The reaction was stopped after 15 min of incubation at room temperature (RT) by the addition of neutralization solution. Then, samples were mixed with 2-mercaptoethanol (5% v/v) and subjected to 12% SDS-PAGE. After electrophoresis, proteins were transferred to nitrocellulose membrane and incubated with rabbit anti-DNP (1:150) followed by a goat anti-rabbit IgG conjugated to horseradish peroxidase (1:300) for 1h at RT, in both cases. To control lane loading, β-actin expression was determined upon membrane incubation with anti-β-actin 1:5000 (8227, Abcam, UK) and anti-rabbit-HRP 1:8000 (ALI0404, Life Technologies), for 1h at RT, in both cases. Chemiluminescent signal was detected upon incubation with ECL, using a ChemiDoc XRS+ System (Biorad). Densitometric analysis was performed with the Image Lab software (Biorad).

**Lipid peroxidation assessment**

Liver samples were fixated in 4% buffered paraformaldehyde pH 7.4 and embedded in paraffin. Tissue sections (5 μm) were adhered to poly-L-lysine treated slides, deparaffinised in xylol and re-hydrated. Tissues were permeabilized for 5 min with the working solution PBS-TritonX-100 0.1%–Tween20 0.1% and the antigen retrieval was performed in 10 mM citrate buffer pH 6.0 by the microwave method (4 x 5 min, 350 watts). After blocking with working solution-BSA 5% for 1h at room temperature (RT), the tissues were washed for 5 min in PBS and treated with MOM IgG Blocking Reagent (Vector MOM Immunodetection kit, Vector Laboratories Ltd., UK) for 1h at RT. Then, samples were incubated with a mouse anti-4HNE antibody 1:50 (MC1019, HNEJ-2, Kamiya Biomedical Company, Seattle, WA, USA) for 1h at RT in MOM diluent solution and subsequently washed with PBS (3 x 5 min). Tissues were incubated with anti-mouse IgG conjugated to Alexa 488 1:500 (A11029, Molecular Probes, Eugene, OR, USA) in MOM diluent solution for 1h at RT. After washing in PBS for 30 min at 4ºC, the tissues were incubated for 15 min in a solution of 0.2 μg/mL DAPI (Sigma) and were mounted with VectaShield (Vector Laboratories). The images were obtained with a Zeiss Axioskop Fluorescence microscope (at a magnification of 400x) and analysed with the Zeiss AxioVision Rel. 4.8.2 software (Carl Zeiss Microscopy GmbH, Germany). Background was subtracted with Photoshop CS5 (Adobe Systems Inc., San Jose, CA, USA). No significant signal was observed in the negative controls (no antibodies; no primary antibody).

**DNA damage assessment**

##### Liver samples were fixated in 4% buffered paraformaldehyde pH 7.4 and embedded in paraffin. Tissue sections (5 μm) were adhered to poly-L-lysine treated slides, deparaffinised in xylol and re-hydrated. Tissues were permeabilized for 5 min with the working solution PBS-TritonX-100 0.1%–Tween20 0.1% and the antigen retrieval was performed by incubating samples with Proteinase K 20 μg/mL in Tris-EDTA-CaCl_2_ buffer for 10 min at 37ºC. Samples were subsequently washed in PBS-Tween 20 0.1% (2 x 5 min). The positive control was treated with DNase I (Life Technologies) for 10 min at RT. Fragmented DNA in all samples was detected through a transferase-mediated dUTP nick-end labeling (TUNEL) staining, according to the instructions of the *In situ* cell death detection kit (Roche). The images were obtained with a Zeiss Axioskop Fluorescence microscope (at a magnification of 400x) and analysed with the Zeiss AxioVision Rel. 4.8.2 software (Carl Zeiss Microscopy GmbH, Germany). Background was subtracted with Photoshop CS5 (Adobe Systems Inc., San Jose, CA, USA). No significant signal was observed in the negative control (no TUNEL reaction mixture, i.e., without terminal transferase).
